# Improving diversity and research inclusion in trials ‐ the impact of ethnic community research champions on recruitment to a study investigating a web‐based assessment of cognition (CognoSpeak)

**DOI:** 10.1002/alz70858_103006

**Published:** 2025-12-24

**Authors:** Dorota Anna Braun, Caitlin H Illingworth, Sahra Abdi, Muse Jama, Nur Ali, Tanya Basharat, Candice Wang, Sarah Ng, Sai Lee, Mohammed Akhlak Rauf, Aneela Nadeem, Lise Sproson, Daniel J. Blackburn

**Affiliations:** ^1^ University of Sheffield, Sheffield, South Yorkshire, United Kingdom; ^2^ Israac Somali Community Association, Sheffield, South Yorkshire, United Kingdom; ^3^ ShipShape Community Hub, Sheffield, South Yorkshire, United Kingdom; ^4^ Sheffield Chinese Community Centre, Sheffield, South Yorkshire, United Kingdom; ^5^ Meri Yaadain CiC, Sheffield, South Yorkshire, United Kingdom; ^6^ NIHR Health Research Centre in Long Term Conditions ‐ Devices for Dignity for Dignity, Sheffield, United Kingdom

## Abstract

**Background:**

Individuals from ethnic minorities are consistently underrepresented in dementia research, which may result in a bias and inadequacy of current measures for these populations. Inclusion of participants from ethnic minorities and those who speak English as a Second Language is especially important in research and development of Artificial Intelligence (AI) based solutions, where existing societal biases are at risk of being amplified.

**Method:**

This paper aims to reflect on recruitment strategies implemented in the development of an AI‐based cognitive assessment tool (CognoSpeak) for recruitment of participants from ethnic minorities. Online recruitment settings proved insufficient in increasing diversity efforts, with participants from an ethnic minority constituting less than 5% of total recruits, despite this recruitment site accounting for over half of all study participants. Similarly, ethnic minority patients recruited through primary and secondary care settings contributed only 1% to total recruitment numbers.

**Result:**

The NIHR PPIE grant allowed for improved diversity and research inclusion. Implementing a participatory approach, including community champions and conducting recruitment in the community centres, rather than traditional research settings allowed for a change of perspective on healthcare and research participation in these groups. The study recruited a total of 152 participants from community groups to date, contributing to 9% of all participants recruited, and 35.4% of all study participants that identified as ethnic minority.

**Conclusion:**

The strategies implemented through the NIHR PPIE grant demonstrate the potential for an increased participation of underrepresented groups in research and should be integrated into traditional recruitment practices to ensure inclusivity and equity across healthcare and technology research.

